# Multiparametric magnetic resonance imaging ultrasound-guided fusion biopsy during active surveillance: A single-centre study

**DOI:** 10.1080/2090598X.2020.1749477

**Published:** 2020-04-17

**Authors:** Kilian Röthlin, Stefania Zamboni, Marco Moschini, Patrick Stucki, Luca Afferi, Philipp Baumeister, Agostino Mattei

**Affiliations:** Department of Urology, Luzerner Kantonsspital, Lucerne, Switzerland

**Keywords:** Prostate cancer, multiparametric magnetic resonance imaging/ultrasound fusion biopsy, 12-core template (standard) biopsy, active surveillance

## Abstract

**Objective:**

To analyse the role of multiparametric magnetic resonance imaging (mpMRI) ultrasound (US)-guided fusion biopsy (FB) in patients with low-risk prostate cancer (PCa) under active surveillance (AS).

**Patients and methods:**

Our retrospective study included 47 patients under AS who consecutively underwent both FB and standard biopsy (SB), from May 2015 until November 2017. We defined FB as a transrectal US-guided biopsy based on mpMRI. The primary endpoint was to assess the rate of concordance between FB and SB in terms of diagnostic yield, as well as the rate of Gleason Score upgrading/downgrading between the two techniques. Cohen’s kappa coefficient (κ) was applied to test the concordance between FB and SB.

**Results:**

The median (interquartile range [IQR]) follow-up was 20 (13–37) months. The median (IQR) number of cores taken was 13 (12–14) at SB and 4 (4–6) at FB. Overall, FB missed 12/47 (26%) PCa diagnoses compared to SB. There was concordance between SB and FB in 64% of the patients. The κ showed a perfect agreement between SB and FB for the detection of PCa with Gleason Score 4 + 4 and a weak concordance for negative biopsies (κ: 0.46) and for PCa with a Gleason Score 4 + 3 (κ: 0.54). There was Gleason Score upgrading at FB in two of 47 (4%) patients, whereas there was downgrading in three of 47 (6%) patients.

**Conclusion:**

In our present study, FB showed no superiority over SB for the detection of PCa. On the contrary, FB had a high rate of missed PCa compared to SB. Further studies are required to ascertain the role of FB in AS.

**Abbreviations:**

AS: active surveillance; FB: fusion biopsy; IL: index lesion; IQR: interquartile range; mpMRI: multiparametric MRI; (cs)PCa: (clinically significant) prostate cancer; PI-RADS: Prostate Imaging-Reporting and Data System; PRIAS: Prostate Cancer Research International Active Surveillance; ROI: region of interest; SB: standard biopsy

## Introduction

Prostate cancer (PCa) is the most commonly diagnosed cancer in USA men, with an estimated 164 690 new cases in 2018 [1]. With the introduction of PSA screening programmes, PCa incidence increased and this led to an important debate about over-diagnosis and consequent potential over-treatment of PCa. Active surveillance (AS) has been proven to be a safe and effective strategy [2–4] for patients with low-risk PCa [5,6]. Although 12-core TRUS-guided standard biopsy (SB) currently remains the ‘gold standard’ for diagnosing PCa [7], this technique samples ~1% of prostate tissue. Consequently, the diagnostic accuracy of this procedure is low and 10–40% of patients on AS have been upstaged by confirmatory targeted biopsies after initial TRUS-guided random SB [8–10].

The progress in multiparametric MRI (mpMRI) allows high-quality images of the prostate and improved PCa identification. The recently released PRECISION trial [11] supported the utility of mpMRI before biopsy and the superiority of mpMRI-targeted biopsy over the 12-core TRUS-guided SB in diagnosing PCa in biopsy naïve men at clinical risk of PCa. Several retrospective series support the utility of mpMRI-based targeted-fusion biopsies (FBs) in detecting clinically significant PCa (csPCa) [12–16]. Moreover, a recent Cochrane Review [17] analysed the role of mpMRI in the repeat-biopsy setting, with a pooled sensitivity of 0.91 (95% CI 0.83–0.95) and a pooled specificity of 0.37 (95% CI 0.29–0.65) for International Society of Urological Pathology (ISUP) Grade ≥2 PCas, although its role in AS is still under debate. For these reasons, the aim of our present study was to analyse the impact and role of FB in patients with low-risk PCa under AS.

## Patients and methods

A total of 345 consecutive patients underwent both mpMRI/TRUS FB and SB in the same session, from May 2015 to November 2017, in a single tertiary care referral centre. A total of 47 patients were identified with low-risk PCa who underwent AS, and thus were included in the study. All the biopsies were taken by a single experienced urologist. Unless otherwise indicated by the patient, biopsies were taken under loco-regional anaesthesia.

We defined SB as all TRUS-guided biopsies taken with a standardised sequence, i.e., six prostatic cores taken from each side of the prostate, as previously described [18]. We defined FB as every TRUS-guided biopsy taken based on previously mpMRI-defined prostatic lesions, i.e., regions of interest (ROIs) at mpMRI of the prostate. FB consisted of at least one biopsy taken from every ROI. All the FBs were performed with the Artemis/Profuse® (Eigen, Grass Valley, CA, USA) platform. The biopsy sequence consisted of SB followed by FB for all the patients included in this study. The reason for this is that we tried to avoid the operator from being influenced to using the same biopsy track of the FB when performing the SB, as this could have allegedly altered the diagnostic yield of the SB [19].

The mpMRI was performed with a 3.0-T scanner (Achieva dStream, Philips Medical Systems, Best, the Netherlands). Most of the mpMRIs were done at our institution and were interpreted by a dedicated genitourinary radiologist, who was previously trained in the reading of mpMRI and who had 2 years’ experience in the assessment of the Prostate Imaging Reporting and Data System (PI-RADS) score at study commencement. The mpMRIs performed in other centres were reviewed at our institution. The PI-RADS score [20] was used for grading the ROIs on the mpMRI. The index lesion (IL) was defined as the ROI with the highest PI-RADS score. If there was an equal PI-RADS score, the ROI with the larger diameter was defined as the IL. All the histopathological analyses were done at our institution and interpreted by dedicated genitourinary histopathologists according to the Swiss Society for Pathology guidelines [21].

### AS

Of the 47 patients included in our study, 38 presented with criteria for inclusion in AS according to the Prostate Cancer Research International Active Surveillance (PRIAS) study: clinical stage T1/T2 PCa, PSA level ≤10 ng/mL, PSA density <0.2 ng/mL/mL, one or two positive biopsy cores, and Gleason Score ≤6 [22]. The remaining nine had low-risk PCa defined as Gleason Score ≤6 and clinical stage T1/T2, but did not strictly adhere to all the PRIAS inclusion criteria. Further we defined csPCa as Gleason Score ≥3 + 4. Monitoring consisted of PSA measurements every 3 months during the first year after biopsy and every 6 months in the second year. Re-biopsies during AS were done annually, if not wished otherwise by the patients. The first biopsy after the enrolment in AS was the confirmatory biopsy, with those following considered as repeat biopsies.

### Outcomes of interest

The primary endpoint was to assess the rate of concordance between FB and SB in terms of diagnostic yield, as well as the rate of Gleason Score upgrading/downgrading between the two techniques. Moreover, we assessed the relationship between the PI-RADS score of the IL and the Gleason Score of the IL at FB, and we looked for predictors of missed PCa at FB. We used the results of the SB as a measure to define missed or not missed PCa diagnosis at FB and vice versa.

### Statistical analysis

Descriptive statistics of categorical variables focused on frequencies and proportions. Means, medians, and interquartile ranges (IQRs) were reported for continuously coded variables. The Cohen’s kappa coefficient (κ) was applied to evaluate the concordance between the results of SB and FB. A univariable logistic regression model was used to assess the relationship between independent variables and upstaging at FB compared to SB. Statistical significance was considered at *P* < 0.05. Statistical analyses were performed using Stata 14 (Stata Corp., College Station, TX, USA).

## Results

### Baseline characteristics

Baseline characteristics of the 47 patients enrolled in AS are reported in Table 1. The median (IQR) follow-up from the first diagnosis of PCa until the last study biopsy was 20 (13–37) months. At the time of confirmatory biopsy, the median (IQR) patient age was 64 (60–68) years, the median (IQR) PSA level was 5.67 (3.90–7.73) ng/mL, and the median (IQR) prostate volume was 50 (33–58) mL. Overall, six patients (13%) at the mpMRI harboured an IL with a PI-RADS score 1, 18 (38%) an IL with a PI-RADS score 2, 12 (26%) an IL with a PI-RADS score 3, nine (19%) an IL with a PI-RADS score 4, and two (4.2%) an IL with a PI-RADS score 5. The median (IQR) IL diameter was 12 (9–17) mm.

**Table 1.** Baseline characteristics of the 47 AS patients.

Variable	Value
Age, years Mean Median (IQR)	63.5 64 (60–68)
PSA, ng/mL Mean Median (IQR)	6.17 5.67 (3.9–7.73)
PSA density, ng/mL/mL Mean Median (IQR)	0.14 0.13 (0.10–0.14)
Prostate volume, mL Mean Median (IQR)	47 50 (33–58)
Number of previous biopsies Mean Median (IQR)	2 2 (1–3)
GS previous biopsies, *n* (%) Negative GS 3 + 3	14 (30) 33 (70)
Suspicious DRE, *n* (%)	4 (14)
Clinical T stage, *n* (%) Negative cT1a cT1b cT1 c cT2	14 (30) 6 (13) 0 24 (51) 3 (6.4)
mpMRI suspicious score, *n* (%) PI-RADS 1 PI-RADS 2 PI-RADS 3 PI-RADS 4 PI-RADS 5	6 (13) 18 (38) 12 (26) 9 (19) 2 (4.2)
Diameter index lesion, mm Mean Median (IQR)	14 12 (9–17)
Time between mpMRI and biopsy, days Mean Median (IQR)	63 42.5 (30–84)

GS, Gleason Score.

### Biopsy results

Biopsy results are reported in Table 2. The median (IQR) number of biopsy cores taken was 13 (12–14) at SB and 4 (4–6) at FB. The FB detected PCa in 15 (32%) patients, whereas the SB detected PCa in 26 (55%) patients. At FB the IL was positive for PCa in 10 (21%) patients.

**Table 2.** The FB and SB results in the 47 AS patients.

Variable	SB	FB
Number of biopsy cores Mean Median (IQR)	13 13 (12–14)	5 4 (4–6)
Number of positive biopsy cores Mean Median (IQR)	1 1 (0–2)	1 0 (0–1)
Number of patients diagnosed with PCa, *n* (%)	26 (55)	15 (32)
Extend of tumor involvement per biopsy core (%) Mean Median (IQR)	11 5 (2–15)	26 25 (4–40)
Positive IL, *n* (%)	–	10 (21)

### Concordance between FB and SB, upgrading/downgrading of Gleason Score at FB

There was concordance between SB and FB in 30/47 patients (64%). Cohen’s κ (Table 3) showed perfect agreement between SB and FB for the detection of PCa Gleason Score 4 + 4 and a weak concordance for negative biopsies (κ: 0.46) and for PCa Gleason Score 4 + 3 (κ: 0.54). There was upgrading of the Gleason Score at FB in two (4%) of the 47 patients, whereas there was downgrading in three (6%). FB and SB missed five of 10 and one of 10 patients with csPCa, respectively.

**Table 3.** Number of cases graded at SB and at concurrent FB and Cohen’s κ assessing the concordance between SB and FB for the 47 AS patients.

		mpMRI/FB, *n*							
		Negative	GS 3 + 3	GS 3 + 4	GS 4 + 3	GS 4 + 4	GS 4 + 5	GS 5 + 4	Total
SB, *n*	Negative	20 κ 0.46	1	0	0	0	–	–	21
	GS 3 + 3	10	6 κ 0.24	0	1	0	–	–	17
	GS 3 + 4	2	2	1 κ 0.24	0	0	–	–	5
	GS 4 + 3	0	1	0	2 κ 0.54	0	–	–	3
	GS 4 + 4	0	0	0	0	1 κ 1	–	–	1
	GS 4 + 5	–	–	–	–	–	–	–	–
	GS 5 + 4	–	–	–	–	–	–	–	–
	Total	32	10	1	3	1	–	–	–

GS, Gleason Score.

### Relationship between the IL PI-RADS score and the Gleason Score of the IL at FB

**Figure 1.**
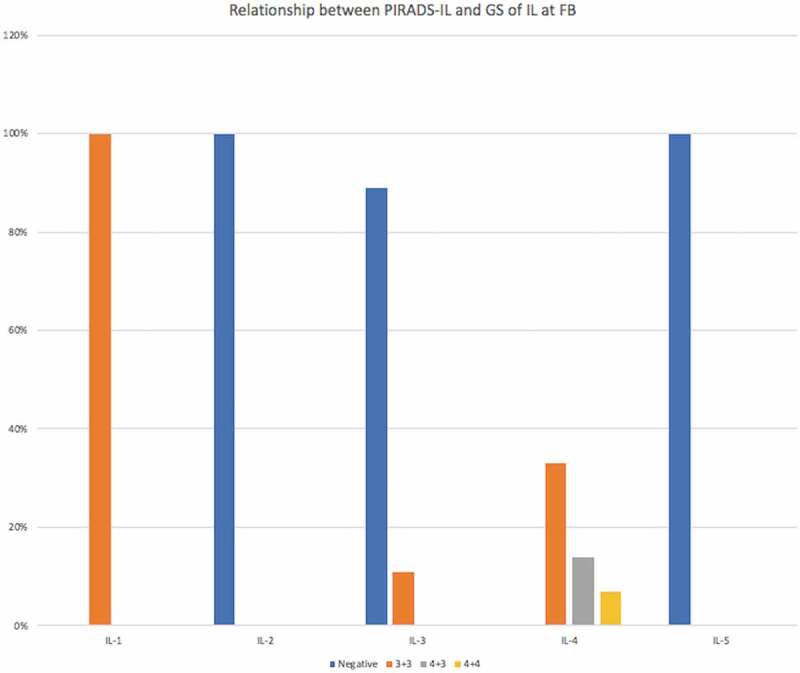
Relationship between PI-RADS score and Gleason-Score (GS) of the (IL) in the 47 AS patients.

The relationship between the IL PI-RADS score and Gleason Score is reported in Figure 1. Only one patient was diagnosed with an IL PI-RADS score 1; he had a Gleason Score 3 + 3 at FB. The four patients with an IL PI-RADS score 2 and the one with an IL PI-RADS score 5 had negative FBs, whereas two of the 18 patients with an IL PI-RADS score 3 had a Gleason Score 3 + 3; the remaining 16 had negative FBs. Of the 15 patients with an IL PI-RADS score 4, one had a Gleason Score 4 + 4, two a Gleason Score 4 + 3, five a Gleason Score 3 + 3, and seven negative FBs.

### Predictors of missed PCa at FB

In our present AS patient cohort, none of the factors tested at univariable analyses were predictors of missed PCa at FB (all *P* > 0.05). Results are reported in Table 4.

**Table 4.** Univariable logistic regression analysis assessing the predictors of missed PCa at FB.

Variable	OR (95% CI)	*P*
Age, years	1.00 (0.89–1.12)	0.9
PSA level, ng/mL	1.00 (0.88–1.14)	0.9
Prostate volume, mL	0.98 (0.93–1.03)	0.5
Positive DRE	3.16 (0.36–27.5)	0.3
Median diameter of IL, mm	0.77 (0.55–1.08)	0.1

OR, odds ratio.

## Discussion

Although FB has been found to be superior to the 12-core TRUS-guided SB in diagnosing PCa in biopsy naïve men at risk of csPCa, sparse data exists regarding its specific role in AS patients. For this reason, we sought to analyse the impact and role of FB in patients with low-risk PCa under AS. Our primary endpoint was to assess the concordance between FB and SB and the rate of missed PCa diagnoses at FB. We found that FB missed 26% of PCa diagnoses. Furthermore, FB missed five of 10 csPCa, whereas SB missed only one of them. This result is consistent with that reported by Ma et al. [23], who found a rate of missed csPCa for FB of 65% and 19% for SB. Two other studies that analysed confirmatory biopsies in AS made similar observations concerning missed csPCa rates [24,25]. On the contrary, multiple studies have reported the superiority of FB over SB in detecting csPCa and reduced missing rates in the diagnostic setting [16–18]. However, in these investigations the whole cohort was divided into two different study arms, specifically patients who underwent either SB + FB or FB + SB were evaluated by two different blinded operators. On the contrary, in our present study the operator was not blinded to the mpMRI results, as the SB was always taken before the FB it is possible that the surgeon took more biopsies during SB near the known ROIs. The diverging results between our present study and those mentioned above could be related to the difference in the study designs.

In our present study, only the ILs with PI-RADS scores ≥4 were associated with the detection of PCa with Gleason Scores ≥3 + 4 at FB. So, it may be possible to omit FB in AS patients with ILs with PI-RADS scores of ≤3 on mpMRI without missing csPCa. However, this finding cannot be generalised to all the PI-RADS score ≤3 cases due to the few patients found with this type of lesion. Moreover, the necessity of taking FB from PI-RADS score 3 lesions has not yet been clarified [21]; to this end, the PRECISION trial avoided taking cores for ROI with PI-RADS scores of <3 [11]. According to Hauth et al. [26], it would be reasonable and practicable to carry out a mpMRI-based follow-up for PI-RADS score 3 lesions instead of taking biopsies in a diagnostic setting. In our present study, we did not find any predictors of missed PCa at FB. Unfortunately, due to the small study population, our logarithmic correlation analysis was only performed with univariable analysis.

Our present study had several limitations, which are mainly related to its retrospective nature. Secondly, the SB and FB were performed by the same urologist, who was not blinded to the distribution and to the PI-RADS scores of the patients’ ROIs; this knowledge may have influenced his choice regarding the core taking in the SB. Thirdly, our AS population was small and heterogeneous: a quarter of patients did not strictly meet AS criteria according to the PRIAS study [22]. Furthermore, because of the small number of patients analysed, it was not possible to calculate a multivariable logarithmic regression. Lastly, another weakness was the comparison of FB and SB against each other, instead of comparing the two biopsy methods with the gold standard of a prostatectomy specimen.

## Conclusion

According to our present study, FB was characterised by a high rate of missed PCa compared to the SB in AS patients. Further prospective studies are required to ascertain the role of FB in patients with PCa under AS.
